# Field-Portable Pixel Super-Resolution Colour Microscope

**DOI:** 10.1371/journal.pone.0076475

**Published:** 2013-09-27

**Authors:** Alon Greenbaum, Najva Akbari, Alborz Feizi, Wei Luo, Aydogan Ozcan

**Affiliations:** 1 Electrical Engineering Department, University of California Los Angeles, Los Angeles, California, United States of America; 2 Bioengineering Department, University of California Los Angeles, Los Angeles, California, United States of America; 3 California NanoSystems Institute, University of California Los Angeles, Los Angeles, California, United States of America; 4 Department of Surgery, School of Medicine, University of California Los Angeles, Los Angeles, California, United States of America; University of Navarra, Spain

## Abstract

Based on partially-coherent digital in-line holography, we report a field-portable microscope that can render lensfree colour images over a wide field-of-view of e.g., >20 mm^2^. This computational holographic microscope weighs less than 145 grams with dimensions smaller than 17×6×5 cm, making it especially suitable for field settings and point-of-care use. In this lensfree imaging design, we merged a colorization algorithm with a source shifting based multi-height pixel super-resolution technique to mitigate ‘rainbow’ like colour artefacts that are typical in holographic imaging. This image processing scheme is based on transforming the colour components of an RGB image into YUV colour space, which separates colour information from brightness component of an image. The resolution of our super-resolution colour microscope was characterized using a USAF test chart to confirm sub-micron spatial resolution, even for reconstructions that employ multi-height phase recovery to handle dense and connected objects. To further demonstrate the performance of this colour microscope Papanicolaou (Pap) smears were also successfully imaged. This field-portable and wide-field computational colour microscope could be useful for tele-medicine applications in resource poor settings.

## Introduction

In recent years there has been a large interest in point of care (POC) imaging and sensing devices that could permit remote clinics or doctors’ offices to conduct basic diagnostic tests that are traditionally restricted to hospitals, requiring a relatively large capital investment [1]–[10]. Recent progress in this direction created various field-portable and cost-effective designs that have the capacity to perform e.g., flow-cytometry [11], optical coherence tomography [12], [13], water quality monitoring [14], fluorescent imaging [15]–[17], and other imaging/sensing tasks [18], [19] even in resource limited settings.

Lensfree holographic on-chip microscopes form an emerging sub-group of such POC imaging devices, and of digital holography in general [20]–[31], offering competitive alternatives to conventional lens-based microscopes, with several key advantages such as wide field-of-view (FOV) and large depth-of-field, yielding giga-pixel range phase and amplitude images, in addition to compactness and cost-effectiveness, making them especially appealing for global health related applications [32]–[35].

These computational microscopes do not use any lenses between the object and the image sensor chip, where the object is placed in close proximity (∼ 0.1–1 mm) to the active area of the sensor chip. A partially coherent light source, e.g., a light emitting diode (LED) is positioned a few centimetres (∼ 5–7 cm) away from the object plane, and the transmitted light pattern through the object is sampled by the sensor chip [32], [36]. Consequently, in this on-chip imaging geometry rather than directly acquiring an image of the object, the diffracted light that is transmitted through the specimen interferes with the unperturbed light from the illumination source, forming an in-line hologram on the image sensor plane. As a result of its unit magnification, the imaging FOV is equivalent to the active area of the image sensor chip, which can be as large as e.g., 20–30 mm^2^ for a Complementary Metal-Oxide-Semiconductor (CMOS) sensor chip or >10 cm^2^ for a Charge-Coupled Device (CCD). Accordingly, the FOV of lensfree on-chip microscopy is significantly wider than the FOV of a lens-based transmission microscope that has a similar resolution level [32], [37]. An additional advantage of these holographic on-chip microscopes is that the recorded image can be digitally focused to different depths, thus allowing screening of large sample volumes [38]–[40].

On the other hand, spatial resolution of such lensfree on-chip microscopes is limited by the pixel size of the image sensor chip. To digitally circumvent this limitation, and hence improve the spatial resolution of these computational microscopes, pixel-super resolution techniques can be utilized by source shifting, which creates sub-pixel shifted replicas of the same object hologram on the sensor-array. This pixel super-resolution approach enables us to reduce the effective pixel-size of the image sensor to digitally enhance the numerical aperture (NA) of the imaging system by e.g., 3 fold, regardless of the sensor type that is used, whether it is a CMOS or CCD imager chip [41].

**Figure 1:**
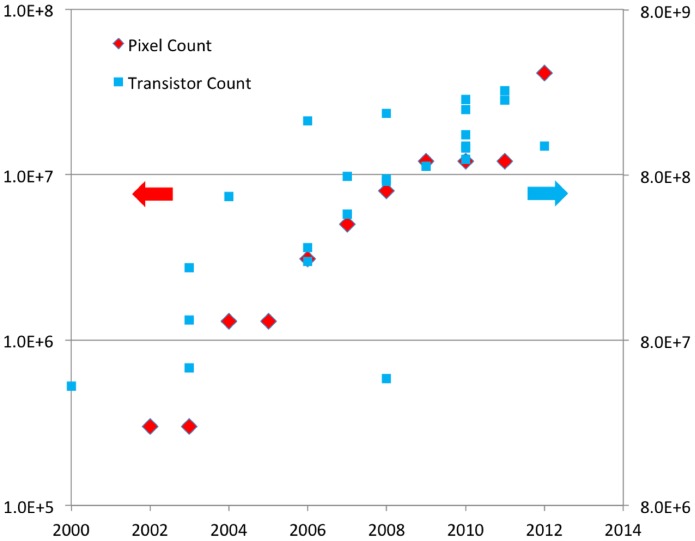
A different view of Moore’s law. A comparison of transistor counts in central processing units (CPUs) versus the pixel counts on cellular phone cameras. The transistor count has several data points for each year, while the cellular phone pixel count has only one data point, which corresponds to the maximum pixel count introduced in that year.

Quite interestingly, these features of lensfree on-chip microscopy are bound to further improve as new opto-electronic sensors are created, mostly driven by consumer electronics market, and in particular cellular phones. Smaller pixel opto-electronic chips with more megapixels that are constantly being introduced on cellular phone cameras will continue to improve the performance of lensfree on-chip microscopes by providing better resolution and/or larger FOV, breaking the traditional trade-off between resolution and FOV of conventional lens-based optical microscopy [32]. As a matter of fact, the pixel count of cellular phone cameras over the last decade has been following Moore’s Law (see Fig. 1), which empirically predicts doubling of the transistor count in Central Processing Units (CPUs) almost every 2 years. Stated differently, since their large-scale introduction in 2002, camera phones have continued to double their megapixel count almost every 2 years, reaching > 40 mega-pixels by the end of 2012. As a direct result of this continued growth, today lensfree imaging can routinely achieve giga-pixel range microscopic phase and amplitude images with an effective NA of e.g., ∼0.8–0.9 across a wide FOV of > 20 mm^2^ [32], [37], [41].

One of the remaining challenges for lensfree field-portable holographic microscopy is high-resolution colour imaging. Colour information in microscopy is rather significant as it serves as a contrast mechanism; for example colour may be used to differentiate different populations of cells using staining such as Trypan blue which is commonly used as a cell viability stain [42]. In addition to this, colour also has a psychological effect since cytotechnologists, pathologists and physicians are in general trained to observe specimen in colour.

**Figure 2:**
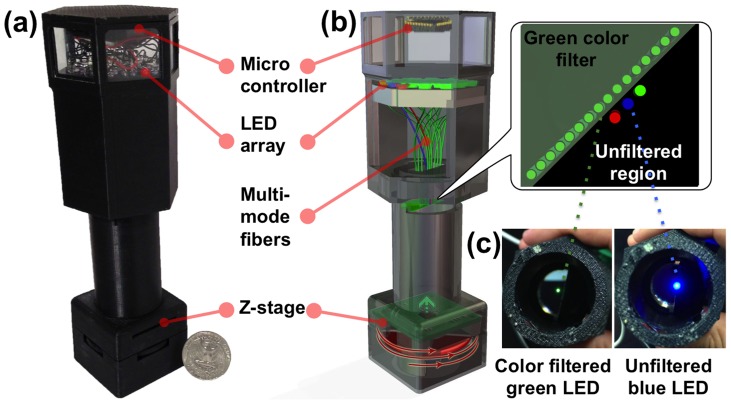
A portable lensfree super-resolution colour microscope. (a) A photograph of the microscope that weighs < 145 grams. (b) A schematic diagram of the microscope; the LED array is separated into two groups, the first group enables pixel super-resolution based on source shifting, and it contains 17 green LEDs (λ  =  527 nm) where each LED is butt-coupled to a multi-mode fiber and its emission passes through a colour filter as shown in the inset. The second group of LEDs enables the acquisition of a lower resolution colour image and it is composed of three LEDs (λ  =  470 nm, 527 nm and 625 nm). (c) A photograph of one of the 17 green LEDs (left) and the blue unfiltered LED (right).

**Figure 3:**
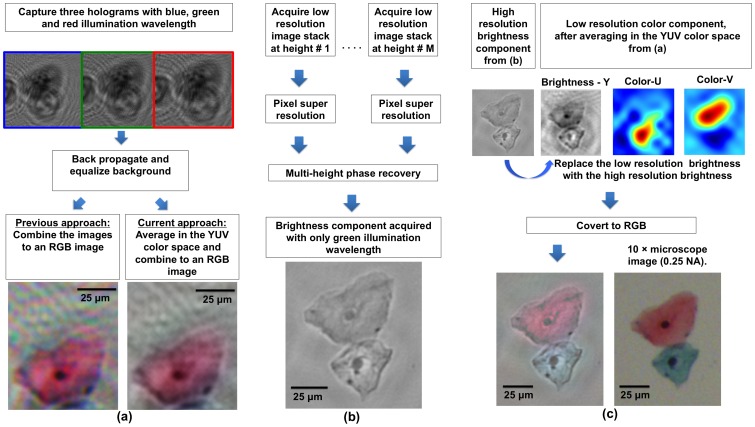
Image processing block diagrams. (a) For creating a lower resolution colour image of the specimen, three lower resolution holograms are acquired, each with a different illumination wavelength (λ  =  470 nm, 527 nm and 625 nm). A previous approach that simply combines these three holograms into a RGB image results in a ‘rainbow’ like colour artefact. The current approach eliminates the colour noise by averaging the colours in the YUV colour space. (b) The flowchart for acquiring and processing a super-resolved multi-height phase-recovery based grey-scale image. (c) A colour image with sub-micron spatial resolution (shown on the left) is rendered by replacing the lower resolution brightness component from (a) with the super-resolved brightness component from (b) in the YUV colour space.

To address this challenge for holographic on-chip microscopy, here we present a lensfree super-resolution colour microscope design, which is cost-effective and field-portable, weighing < 145 grams with dimensions of < 17×6×5 cm (see Fig. 2). In this field-portable colour microscope, we introduce an image processing technique that mitigates ‘rainbow’ like colour artefacts (see e.g., Fig. 3a) that are characteristic of digital holography [43]–[47]. This colorization technique is based on transforming the lensfree RGB image into the YUV colour space [48], [49], which separates the brightness component of the image (i.e., Y channel) from its colour components (i.e., U and V channels). As a result of this, to achieve sub-micron spatial resolution in colour, there is no need to capture three distinct pixel super-resolved holograms, each with a different illumination colour (i.e., red, green and blue). In fact, it is sufficient to acquire only one pixel super-resolved lensfree image with a single illumination wavelength (e.g., green) which can serve as the Y channel of the colour image, preserving the sub-micron resolution over a large FOV. To obtain the remaining U and V channels of the colour image, since there is no need for pixel super-resolution, three LEDs at red, green and blue wavelengths would be sufficient. To demonstrate the resolution and colour imaging capabilities of this field-portable holographic microscope, we imaged a USAF resolution test chart and colour stained Papanicolaou (Pap) smears, which are widely used for pre-screening of cervical cancer [50], [51].

Recently, we also introduced a lensfree colour microscopy concept, which uses similar image processing methods to mitigate ‘rainbow’ like colour artefacts. However, this previous work was performed on a bench-top system within a well-controlled environment, which involved an optical table as well as a mechanical micro-stage and a monochromator to control the illumination position and the wavelength [52]. Here we introduce a novel design and the experimental implementation of a field-portable, light-weight and cost-effective colour microscope, which is based on inexpensive components such as an array of LEDs and 3D printed plastic enclosure. This field-portable, cost-effective and wide-field colour microscope could be significant especially for tele-pathology and micro-biology applications in resource limited environments and developing countries.

## Methods

### Field-portable colour microscope design

Our field-portable microscope (see Fig. 2) is designed to enable both pixel super-resolution and colour imaging by separating a set of low-cost LEDs that serve as the illumination source into two groups. The first group of LEDs enables pixel super-resolution imaging based on a shift of the source location; it contains 17 green LEDs that are butt-coupled to multi-mode fibers (each with 0.1 mm core diameter, Thorlabs, AFS-105/125Y). The emission of these green LEDs is also passed through a colour filter after each fiber end (bandwidth ∼ 3 nm, centred around 532 nm, Thorlabs, FL532-3) to achieve the temporal coherence that is required for capturing high NA in-line holograms at the detector plane. To implement pixel-super resolution by source shifting, these green LEDs of the first group are arranged into a line, which is tilted 45 degrees relative to the image sensor edges (see Fig. 2.b), such that the acquired lensfree holograms can be sub-pixel shifted along both X and Y directions.

The second group of LEDs enables the acquisition of lower resolution lensfree colour images, and it is composed of only three LEDs: one blue, one green, and one red, with λ  =  470 nm, 527 nm and 625 nm, respectively. The illumination bandwidth of each one of these LEDs is rather broad, e.g., ∼ 45 nm, and since pixel super-resolution is not needed here, we did *not* employ a colour filter for these second group of LEDs. Each LED is still butt-coupled to a multi-mode fiber (0.1 mm core diameter), as illustrated in Fig. 2.c.

All these 20 LEDs are individually controlled by a micro-controller (Atmel, ATmega8515), which sequentially turns on and off each LED within the array, while the image sensor captures lensfree holograms. The image capture and illumination are controlled in LabVIEW, and consequently the entire imaging process, including auto-exposure, is fully automated and is controlled using a laptop computer with a USB connection to the field-portable microscope.

The distance between the fibers’ free ends (which are cleaved) and the object plane is designed to be ∼ 6 cm for two reasons: first, to allow the partially-coherent illumination light to gain sufficient spatial coherence before impinging on the sample plane, and second to ensure that each LED will illuminate the sample with an angle that is less than three degrees compared to the surface normal. This restriction is of paramount importance for pixel-super resolution based microscopy, since it enables capturing the same cross-section of the object in each sub-pixel shifted transmission image.

The above described multi-source illumination module is then connected to the sample holder and a cost-effective, custom-designed Z-stage (see Fig. 2.b). This Z-stage has a coarse axial resolution of ∼ 10–15 µm, and it is used to move the CMOS sensor chip (1.67 µm pixel size, 10 mega-pixel, monochrome) up and down compared to the sample plane, which is required for achieving multi-height based phase-recovery. This multi-height phase-recovery process enables imaging of dense and connected samples, such as pathology slides, by iteratively eliminating the twin image noise, which is an artefact of in-line holography. This Z-stage is custom built from a lens-tube (Thorlabs, SM1L03) and a threading adapter (Thorlabs, SM1A10), which is glued to the field-portable microscope enclosure.

### Digital colorization of lensfree holographic images

To obtain high fidelity colour reproduction of the imaged object, three sequential lensfree holograms are initially acquired, each with a different illumination wavelength: blue, green and red (λ  =  470 nm, 527 nm and 625 nm, respectively). Then, the background mean values of these holograms are equalized to digitally compensate for power and fiber coupling efficiency variations between different LEDs in our microscope design. These resulting in-line holograms are then back propagated to the object plane (see Fig. 3.a). If these in-line holograms were simply combined into an RGB image [43], a ‘rainbow’ like colour artefact would corrupt the image [43]–[47]. Therefore, to mitigate this artefact, the lensfree RGB image is initially converted into the YUV colour space using Colorspace Transformations package that is processed in MATLAB. The YUV colour space separates the brightness information (Y channel) from the colour information of the image (U and V channels), and therefore by averaging only the colour components with a rectangular window (∼ 13 µm edge size), the ‘rainbow’ like colour artefact of lensfree holographic images can be mitigated (Fig. 3.a). However, the spatial resolution of the resulting image becomes relatively low, and to enhance its resolution, the brightness component (Y channel) is entirely replaced by the pixel super-resolved image of the object (Fig. 3.b), which is obtained by using 17 green LEDs that are butt-coupled to a linear array of multi-mode fiber optic cables, as detailed earlier. The resulting colour image has the brightness component (Y channel) from Fig. 3.b that is super-resolved, and the colour information (U-V channels) from Fig. 3.a.

To further enhance the contrast of important sub-cellular features such as cell nuclei, which are typically absorbing and characterized by low intensity values in their lensfree transmission images, dark areas within the brightness (Y) channel were not coloured. These dark areas of an image were detected by thresholding the super-resolved brightness image. The resulting ‘hybrid’ YUV image is then converted back to RGB colour space to achieve high fidelity colour representation, as illustrated in Fig. 3.c, which is in very good agreement with a conventional lens-based microscope image of the same sample.

### Source shifting based pixel super-resolution

Pixel super-resolution is a computational method that synthesizes one high-resolution image from a set of sub-pixel shifted lower resolution images of the same scene/object [53]–[58]. The resulting high-resolution image is effectively equivalent to digitizing the object with a smaller pixel-size sensor. In our field-portable microscope, we shift the light source (LED) location by sequentially illuminating the sample with 17 colour filtered LEDs (Fig. 2.b), each of which is butt-coupled to a multi-mode fiber. The sub-pixel shifts between different holograms are evaluated automatically by implementing an iterative gradient method [55]. These relative shifts together with the lower resolution raw holograms are then provided as inputs to a least-square optimization problem to estimate a high-resolution (i.e., super-resolved) hologram that is consistent with the lower resolution measured lensfree holograms, while also penalizing for high-frequency components that might arise due to measurement noise. This optimization problem can be quickly solved by a conjugate gradient method [53], which typically converges within ∼10 iterations.

### Multi-height phase-recovery

A well-known artefact of in-line holography is the twin image noise, which corrupts the reconstructed holograms especially for spatially dense and connected samples [59]. To mitigate this twin image noise, various phase-recovery approaches were devised [36], [59]. In this work, we implemented a multi-height phase-recovery approach since it does not need prior knowledge about the object shape or dimensions [60]–[64]. This iterative phase recovery approach uses few intensity measurements (e.g., 3–4), where each measurement is obtained at a different sample to sensor distance (i.e., height). The multi-height phase-recovery approach propagates the super-resolved holograms between different heights, and at each step the algorithm enforces the super-resolved hologram intensity, while keeping the resulting phase from the previous iteration untouched. After several iterations (e.g., 5–10) the missing phase is retrieved and the twin image noise is significantly suppressed. In our implementation, to change the distance between the image sensor and the sample planes, a cost-effective and custom-designed Z-stage was used (see Fig. 2). In case the sample is too thick to fit into the insertion tray of our microscope, various glass cover slips with different thicknesses can also be placed between the sample and the image sensor to change the sample’s height compared to the sensor chip. An auto-focus algorithm was also implemented to automatically estimate the sample to sensor distance for each raw hologram without the need for an independent measurement of the sample height [14].

### Hologram reconstruction - focusing back to the object plane

After the phase recovery steps described earlier, the final holographic reconstruction process includes the multiplication of the complex hologram with a reference wave, which in our imaging geometry can be approximated as a plane wave. The hologram can then be back propagated to the object plane by using the free space transfer function in the frequency domain [59]. This computational step results in a complex image, containing both the amplitude and phase information of the objects.

## Results

**Figure 4:**
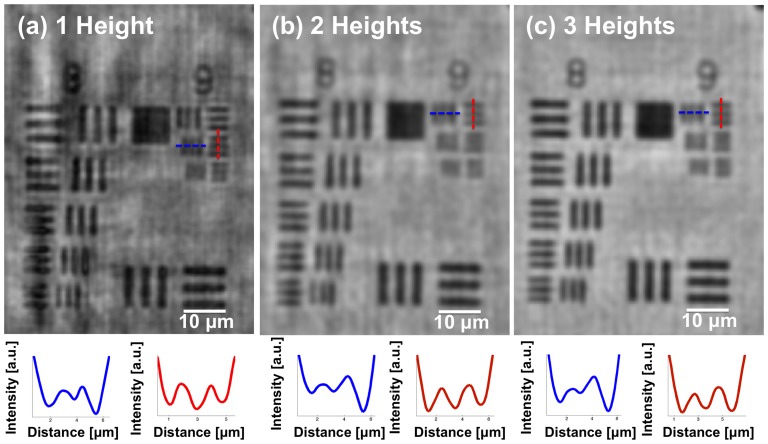
Quantification of the spatial resolution as a function of the number of heights used in our multi-height phase-recovery process. (a) Amplitude image that was reconstructed using one height. (b) Amplitude image that was reconstructed using two heights. (c) Amplitude image that was reconstructed using three heights. Cross sections of the smallest resolved gratings are provided below each reconstructed image. Pixel size of the monochrome CMOS chip used in our field-portable microscope is 1.67 µm. Note that using a CMOS imager chip that has a smaller pixel size (e.g., ∼1.1 µm) [37], a higher spatial resolution of e.g., ∼300 nm can also be achieved using the same lensfree imaging technique [41].

To quantify the spatial resolution of our field-portable microscope as a function of the number of heights used in our multi-height phase-recovery process, a 1951 USAF resolution test chart was imaged. In our image reconstruction process, we followed the computational flowchart shown in Fig. 3.b. Fig. 4.a shows the reconstructed amplitude image using only one height (Z  =  694 µm), which clearly resolves a line-width of 0.87 µm using a CMOS imager chip that has a pixel size of 1.67 µm. Fig. 4.b, on the other hand, shows the reconstructed amplitude image using two different heights (694 µm and 922 µm), which resolves a line-width of 0.98 µm. It is clear that using two different heights, the twin image noise is suppressed in comparison to Fig. 4.a; however, the resolution is also slightly degraded due to possible errors in spatial registration of the two super-resolved holograms acquired at different heights. When using three heights (694 µm, 922 µm and 958 µm), no additional degradation in resolution is observed as illustrated in Fig. 4.c; nevertheless the twin image noise is now significantly cleared (for instance, the dark vertical lines in Fig. 4.a are not present in Fig. 4.c). In these experiments, our goal was to illustrate this significant reduction of twin image related spatial artefacts, while also demonstrating that cross-registration of multi-height super-resolved holograms and their FOVs does not introduce a major resolution loss, still permitting sub-micron resolution as shown in Figs. 4.b and 4.c.

**Figure 5:**
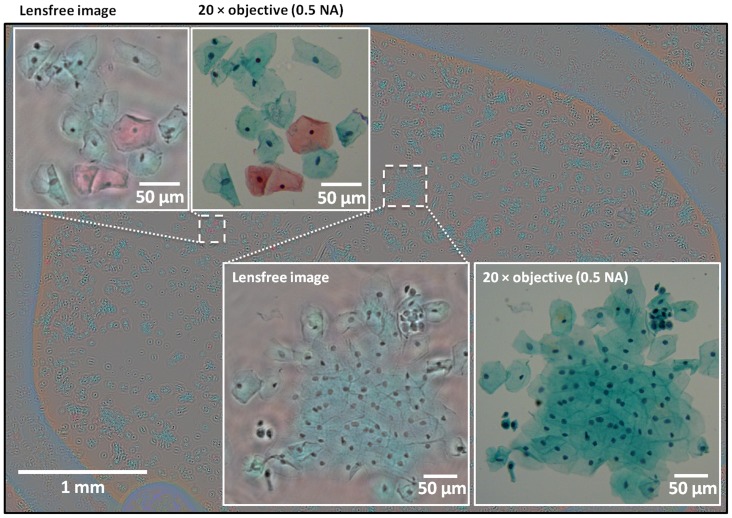
A wide FOV (∼21 mm^2^) lensfree colour image of a Pap smear sample (ThinPrep® preparation). The Pap test was reconstructed using pixel super-resolved holograms acquired at four different heights (1069 µm, 1117 µm, 1159 µm and 1205 µm). For comparison purposes, 20× microscopes images (0.5 NA objective lens) of the same specimen are also provided.

**Figure 6:**
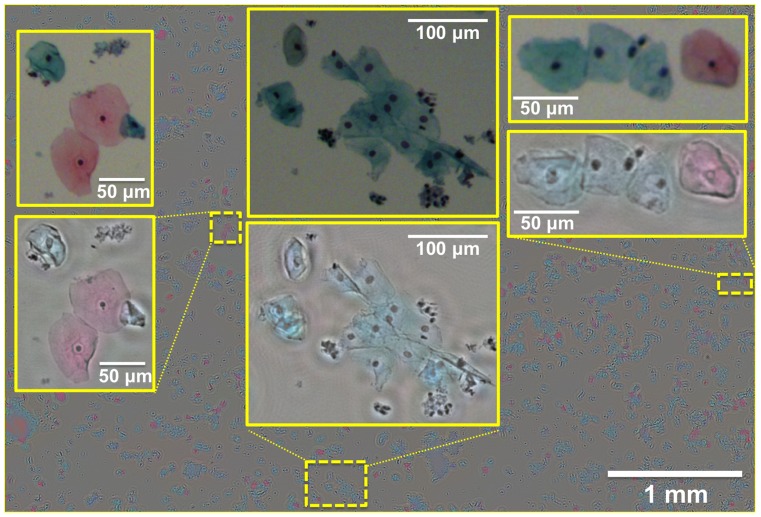
A wide FOV (∼21 mm^2^) lensfree colour image of a Pap smear sample (ThinPrep® preparation). The Pap test was reconstructed using pixel super-resolved holograms acquired at three different heights (860 µm, 1040 µm and 1080 µm). For comparison purposes, 10× microscopes images (0.25 NA objective lens) are also provided, located above their corresponding lensfree colour images.

To further illustrate the performance of our field-portable colour microscope, next we imaged Pap smear samples over a wide FOV of ∼ 21 mm^2^, the results of which are summarized in Figs. 5 and 6. Pap test is a cytology-based method that is extensively used to diagnose cervical cancer, which is the second most common cancer in women worldwide [51]. Optical microscopy can be considered as one of the gold standard techniques for early detection of premalignant and/or malignant cells in a Pap smear, a task that is labour intensive and tedious, since in early stages of cervical cancer only a few cells among thousands of cells will statistically be abnormal. Moreover colour is important in Pap tests since it allows differentiating between different states/types of cells on a Pap smear slide [65]. To create the lensfree colour images shown in Figs. 5 and 6, the computational flowchart of Fig. 3 was utilized, where pixel super-resolved holograms were synthesized at three/four different heights and five multi-height phase-recovery iterations were computed. Our reconstructed lensfree colour images provide very good agreement with 20× microscope objective (0.5 NA) images in Fig. 5 and with 10× microscope objective (0.25 NA) images in Fig. 6, which are provided for comparison purposes. These results demonstrate the potential of this field-portable colour microscope for tele-pathology and micro-biology applications, making it especially relevant for various global health applications.

## Discussion

Since the low-resolution colour image is overlaid on the high-resolution brightness image, it is evident that our microscope has a significantly better resolution in the brightness channel than in the colour channel. Unlike the brightness channel, where the spatial resolution is determined by the effective pixel size of the image sensor after pixel super-resolution, e.g., ≤500 nm, the colour resolution is determined by the size of the averaging window in the YUV colour space (e.g., ∼ 13 µm), which is used to mitigate the rainbow colour artefacts in reconstructed holographic images. However, in the context of Pap tests or pathology in general, this resolution mismatch between the colour (i.e., UV) and brightness (i.e., Y) channels is irrelevant. Sub-micrometer resolution is required in clinical pathology applications to observe the cells’ boundaries and nuclei; this information is provided in the brightness channel of our field-portable microscope. On the other hand, the colour information is used as a visual marker to distinguish among different cell types; therefore lower colour resolution requirements are acceptable. Moreover, the physical process of colour staining creates an inherent resolution loss as the dye molecules diffuse inside the stained cell, creating smoothened boundaries. Consequently, the presented microscopic colorization scheme satisfies pathology needs, as it provides accurate colour information that would have been normally concealed by the rainbow artefacts of holographic imaging.

**Figure 7:**
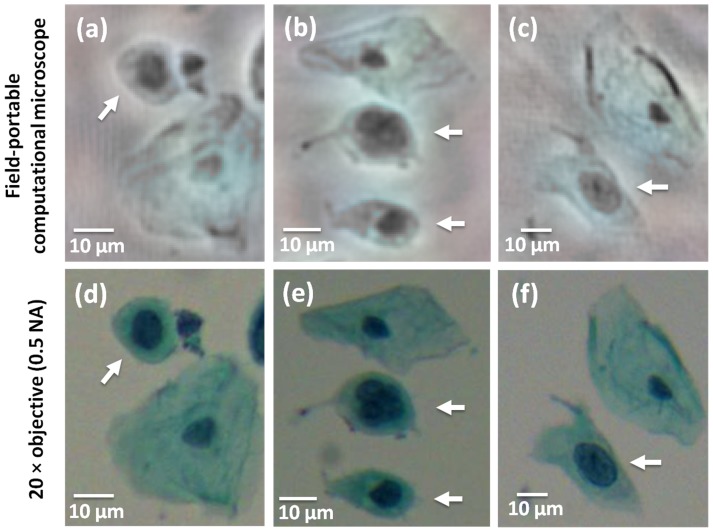
Lensfree computational images of abnormal cells in a Pap smear sample. (a), (b) and (c) lensfree Pap smear images of abnormal cells (pointed by white arrows), which generally exhibit large nucleus in comparison to their cytoplasm as well as an irregular cytoplasm shape. (d), (e) and (f) microscope comparison images for (a), (b) and (c), respectively, obtained using a 20× microscope objective (0.5 NA).

To validate that our field-portable microscope can satisfy pathology needs, we specifically imaged some abnormal cells in a Pap smear as shown in Fig. 7 (marked by white arrows). A professional cytotechnologist confirmed the abnormality of these cells beforehand and the cells were imaged with our field-portable device. Abnormal cells have generally distinct spatial features such as a large nuclear-cytoplasmic ratio and/or an irregular cytoplasm shape. Figures 7.a-c illustrate the lensfree colour images obtained by our field-portable microscope using four different sample heights (i.e., 1069 µm, 1117 µm, 1159 µm and 1205 µm). These lensfree images shown in Figs. 7.a-c agree very well with the corresponding 20 × microscope objective (0.5 NA) images that are provided in Figs. 7.d-f, respectively, illustrating that our field-portable colour microscope preserves the spatial features of interest within abnormal cells. Here we should also emphasize that this work does ‘not’ claim automated diagnosis of Pap smears and does ‘not’ aim to replace the pathologist or the cytotechnologist; but rather aims to create a field-portable and cost-effective high-throughput microscope that can provide decent quality and high-resolution colour images of various specimen that can be used by professionals for e.g., tele-pathology and remote diagnostics purposes.

It is interesting to note that in this computational colour microscope, while the usage of multiple heights offers superior twin image elimination, it also comes with the cost of partially degrading the spatial resolution of the image as well increasing the reconstruction time. Typically, the twin image noise is strong when the specimen is spatially dense and connected, and it gets weaker when the specimen is spatially sparse. Therefore, if sub-micron resolution is sufficient for a certain imaging application/need, the number of heights to be used in the multi-height phase-recovery process depends on the spatial density of the sample of interest. For samples that are spatially dense and/or require the investigation of fine features obscured by twin image related artefacts (as in the case of Pap smear samples, see e.g., Fig. 5), the multi-height phase-recovery process might require more than two different heights in order to produce acceptable results and effectively eliminate the twin image noise. As a solution to this need, our field-portable colour microscope offers the flexibility to acquire super-resolved holograms at any necessary number of sample heights, thus enabling computational colour imaging of dense and connected specimen. Finally, we should emphasize that using a CMOS imager chip that has an even smaller pixel size (e.g., ∼1.1 µm) [37], a higher spatial resolution of ∼ 300 nm can also be achieved using the same computational imaging technique [41].

## Conclusions

We demonstrated a field-portable colour microscope that can achieve sub-micron resolution over a wide FOV of e.g., > 21 mm^2^. Given its light weight (< 145 grams) and small dimensions (< 17 cm×6 cm×5 cm), this computational microscope is especially suitable for field use. This microscope’s design combines pixel super-resolution and multi-height phase-recovery techniques with a colorization algorithm to significantly reduce ‘rainbow’ like artefacts observed in colour holographic imaging. To demonstrate the resolution and colour imaging capabilities of this computational microscope, we imaged a resolution test target and stained Pap smear samples. This light-weight, cost-effective and wide-field colour microscope might be rather useful for tele-pathology and micro-biology applications in resource poor settings and developing countries.
